# MicroRNA 181b Regulates Decorin Production by Dermal Fibroblasts and May Be a Potential Therapy for Hypertrophic Scar

**DOI:** 10.1371/journal.pone.0123054

**Published:** 2015-04-02

**Authors:** Peter Kwan, Jie Ding, Edward E. Tredget

**Affiliations:** 1 Division of Plastic Surgery, Department of Surgery, Faculty of Medicine and Dentistry, University of Alberta, Edmonton, Alberta, Canada; 2 Wound Healing Research Group, Department of Surgery, Faculty of Medicine and Dentistry, University of Alberta, Edmonton, Alberta, Canada; 3 Division of Critical Care, Department of Medicine, University of Alberta Hospital, Edmonton, Alberta, Canada; Medical University of South Carolina, UNITED STATES

## Abstract

Hypertrophic scarring is a frequent fibroproliferative complication following deep dermal burns leading to impaired function and lifelong disfigurement. Decorin reduces fibrosis and induces regeneration in many tissues, and is significantly downregulated in hypertrophic scar and normal deep dermal fibroblasts. It was hypothesized that microRNAs in these fibroblasts downregulate decorin and blocking them would increase decorin and may prevent hypertrophic scarring. Lower decorin levels were found in hypertrophic scar as compared to normal skin, and in deep as compared to superficial dermis. A decorin 3’ un-translated region reporter assay demonstrated microRNA decreased decorin in deep dermal fibroblasts, and microRNA screening predicted miR- 24, 181b, 421, 526b, or 543 as candidates. After finding increased levels of mir-181b in deep dermal fibroblasts, it was demonstrated that TGF-β_1_ stimulation decreased miR-24 but increased miR-181b and that hypertrophic scar and deep dermis contained increased levels of miR-181b. By blocking miR-181b with an antagomiR, it was possible to increase decorin protein expression in dermal fibroblasts. This suggests miR-181b is involved in the differential expression of decorin in skin and wound healing. Furthermore, blocking miR-181b reversed TGF-β_1_ induced decorin downregulation and myofibroblast differentiation in hypertrophic scar fibroblasts, suggesting a potential therapy for hypertrophic scar.

## Introduction

The genetic regulation underlying wound healing and its dysregulation in hypertrophic scar (HSc) is complex and incompletely understood [1, 2]. HSc following burns share many features with fibroproliferative disorders like pulmonary fibrosis, renal fibrosis, and scleroderma [3]. Unfortunately current therapies for HSc are of limited efficacy [4]. Clinically HSc is red, raised, pruritic, and inelastic scar in the original zone of injury [5]. It impairs function [6], and its disfiguring effects can cause lifelong psychosocial morbidity [7]. Histologically, HSc is characterized by increased myofibroblasts and mast cells, hypervascularity, excessive extracellular matrix (ECM) [8], whorls or nodules [9], and significantly decreased decorin (DCN) [10].

DCN is a small, leucine-rich proteoglycan [11] that plays key roles in ECM where it inactivates profibrotic transforming growth factor beta (TGF-β) [12] and connective tissue growth factor (CTGF/CCN2) [13], and antagonizes multiple cell surface receptors, including epidermal growth factor receptor [14], insulin like growth factor 1 receptor [15], and hepatocyte growth factor receptor [16]. In animal models DCN reduces cancer metastases [17], decreases renal [18] and pulmonary [19] fibrosis, improves post-infarction myocardial remodeling [20], and induces spinal cord regeneration [21]. DCN has been proposed as a treatment for HSc based on its in vitro ability to reduce collagen gel contraction by HSc fibroblasts [22], decrease cellular proliferation, reduce TGF-β_1_ production, and decrease collagen synthesis [23]. Previous work demonstrates that DCN is significantly downregulated in HSc versus normal skin (NS) fibroblasts [24], and in deep dermal fibroblasts (DF) versus superficial dermal fibroblasts (SF) [25]. In a linear scratch model of increasing dermal depth Dunkin et al. found superficial injury regenerated and deeper injury scarred [26]. These observations suggest DCN production by SF is important for dermal regeneration and decreased production by DF contributes to scarring. Furthermore, it has been proposed that HSc arises from DF [25,27].

MicroRNA (miRNA) are short, endogenous RNA, predicted to post-transcriptionally regulate approximately two thirds of human protein encoding genes [28]. They bind to the 3’UTR (un-translated region) of mRNA through seed region base pairing and decrease protein expression via effects on mRNA stability or translation [29]. The importance of miRNAs in skin development, homeostasis, and disease has been recently highlighted [30,31], as has their role in fibrosis [32], and regulation of the proteoglycan versican [33].

Our hypothesis is that since miRNA often regulate related cell signaling networks [34], determining ones regulating DCN could indicate miRNA with roles in other fibrotic pathways and provide therapeutic targets with diverse effects. Based on differences between HSc and NS, and DF and SF, it is possible that increased expression of miRNA targeting DCN in HSc and DF might help explain their reduced DCN expression and provide insight into HSc pathophysiology.

## Materials and Methods

### Primary Human Cells and Tissue Specimens

HSc and site-matched NS biopsies from burn patients, and matched SF and DF from human abdominoplasty specimens were obtained with written informed consent under protocols approved by the University of Alberta Hospital Health Research Ethics Board and conducted according to the Declaration of Helsinki Principles (S1 Table). Dermal fibroblasts were cultured from NS and HSc using explanation [35], or from abdominoplasty specimens using a dermatome to separate dermis into superficial and deep layers for enzymatic extraction of fibroblasts [25,35]. Fibroblasts were propagated in Dulbecco’s Modified Eagle Medium (DMEM) (Invitrogen, Carlsbad, CA) supplemented with 10% fetal bovine serum (FBS) (Invitrogen) and antibiotic-antimycotic (Invitrogen) in an incubator at 37°C in atmospheric air with 5% CO_2_. Fibroblasts at passages 3–5 were used.

### DCN Immunohistochemistry

Biopsies of site-matched HSc and NS were fixed in Z-Fix (Anatech Limited, Battle Creek, MI) for 24 hours then processed into paraffin blocks, cut into 5 μm sections, and mounted on glass slides by the Alberta Diabetes Institute Histology Core Laboratory (University of Alberta, Edmonton, Canada). Sections were deparaffinized using sequential xylene and ethanol baths, then blocked with Image-iT FX (Invitrogen), and then 10% goat serum (Jackson ImmunoResearch Laboratories, West Grove, PA) and 1% bovine serum albumin (Sigma-Aldrich Corporation, St. Louis, MO). Sections were incubated at 4°C overnight with primary polyclonal goat anti-human DCN antibody (R&D Systems, Minneapolis, MN) diluted in 1% bovine serum albumin or diluent with antibody omitted as a negative control. Sections were then incubated with a secondary Alexa Fluor 488 chicken anti-goat antibody (Invitrogen) diluted 1:200 at room temperature in the dark for 1 hour. Specimens were mounted in ProLong Gold with DAPI (Invitrogen) under glass cover slips, imaged using a Zeiss Colibri microscope (Carl Zeiss MicroImaging, Thornwood, NY), and fluorescence measured using ImageJ (National Institutes of Health, Bethesda, MD).

### DCN 3’ UTR Reporter Assay

The DCN 3’UTR was cloned from a DCN cDNA (accession # BC005322) plasmid pDNR-LIB-DCN (Open Biosystems Products, Huntsville, AL) and inserted into pCAG-DsRed2 [36] from Addgene plasmid 15777 (Addgene, Cambridge, MA) between the stop codon and poly(A) sequence using Sticky-End PCR [37] to form pCAG-DsRed2-D3U (all primer sequences in S2 Table). pCAG-EmGFP was generated by replacing DsRed2 in pCAG-DsRed2 with EmGFP from pRSET-EmGFP (Invitrogen) using Sticky-End PCR. Plasmids were verified by sequencing at The Applied Genomics Centre (University of Alberta). SF or DF were grown on glass cover slips in DMEM + 10% FBS. Equimolar amounts of pCAG-DsRed2 and pCAG-EmGFP, or pCAG-DsRed2-D3U and pCAG-EmGFP were transfected using Lipofectamine LTX (Invitrogen), according to the manufacturer’s instructions, then cultured for a further 48 hours in DMEM + 2% FBS. Cells were fixed in fresh 2% formaldehyde for 5 minutes, mounted on glass slides in ProLong Gold with DAPI, imaged using a Zeiss Colibri microscope, and relative intensities calculated using ImageJ.

### miRNA Screening

To determine potential miRNA regulating DCN we used prediction algorithms TargetScan [38], and miRanda [39]. Results were manually curated to select miRNA predicted to interact with other wound healing and fibrosis genes.

To further screen miRNA interactions a DCN 3’UTR qPCR screening protocol was developed. Briefly, qPCR primers were designed as follows and ordered from Eurofins MWG Operon (Huntsville, AL). A forward primer was designed with perfect homology to a sequence upstream of the DCN 3’UTR. A positive control reverse primer with perfect homology to a sequence downstream of the DCN 3’UTR and a negative control reverse primer with a scrambled sequence were design. miRNA primers were designed using mature miRNA sequences from miRBase [40]. qPCR was performed with 5 ng of pDNR-LIB-DCN as template, appropriate primers, RT^2^ SYBR Green / ROX qPCR Master Mix (SABiosciences, Frederick, MD). The amplification efficiency of the positive control primers was set to 2.0 and calculations of amplification efficiency for remaining primer combinations were performed. Primer combinations with efficiencies ≥ 1.35 were selected as potential interactions based on acceptable efficiencies [41], and the remainder considered non-interactions.

### RT-qPCR

Total RNA was isolated from cell culture using TRIzol (Invitrogen) according to manufacturer protocols with addition of GlycoBlue (Invitrogen) during isopropanol precipitation. Tissue for RNA extraction was flash frozen in liquid nitrogen, stored at -80°C until it was ground to a fine powder in a chilled pestle and mortar, then dissolved in TRIzol and total RNA was isolated. Total RNA was reverse transcribed to cDNA using miScript (QIAGEN, Valencia, CA). RT-qPCR was performed using RT^2^ SYBR Green / ROX qPCR Master Mix (QIAGEN). RT-qPCR of miRNA was performed using miRNA specific primers (QIAGEN) according to manufacturer protocols and relative expression calculated using the comparative C_T_ method [42] with reference gene RNU6B. RT-qPCR of mRNA was performed using primers listed in S2 Table with reference gene HPRT1.

### TGF-β_1_ and CTGF Stimulation of Dermal Fibroblasts and Measurement of miRNA by RT-qPCR and DCN Protein by ELISA

Matched SF and DF in DMEM + 2% FBS were stimulated with recombinant human TGF-β_1_ (10 and 20 ng/mL) or CTGF (5 and 10 ng/mL) (R&D Systems). Total RNA was harvested and RT-qPCR of miR-24 and miR-181b was performed.

Recombinant human TGF-β_1_ was used to stimulate site-matched NS and HSc fibroblasts in DMEM + 2% FBS at various concentrations for 48 hours. AntagomiR-181b (QIAGEN) was transfected into HSc fibroblasts using HiPerFect (QIAGEN). Cell culture supernatant was collected and DCN was measured using a human DCN ELISA kit (R&D Systems) according to manufacturer protocols.

### Dual Luciferase Reporter Assay to Measure miR-181b Interactions with Potential Binding Sites from DCN 3’UTR

A dual luciferase reporter assay, pmirGLO (Promega, Madison, WI), had potential miRNA binding sites (S3 Table) inserted using the manufacturer’s protocol, to create reporters: pmirGLO-miR181b, pmirGLO-scramble, pmirGLO-DCN1, pmirGLO-DCN2, and pmirGLO-DCN3. Plasmids were verified by sequencing. HEK293A cells (American Type Culture Collection, Manassas, VA) were cultured in a 96 well plate using DMEM + 2% FBS and transfected with pmirGLO, pmirGLO-181b, pmirGLO-scramble, pmirGLO-DCN1, pmirGLO-DCN2, or pmirGLO-DCN3, and synthetic miR-181b (QIAGEN) using HiPerFect. After 48 hours relative luminescence was measured using the Dual-Luciferase Reporter Assay System (Promega) and an EnVision 2104 Multilabel Reader (PerkinElmer, Waltham, MA).

### Synthetic miR-181b, DCN siRNA, and antagomiR-181b Treatment of Dermal Fibroblasts and Measurement of DCN mRNA by RT-qPCR and DCN Protein by ELISA

Untreated, or control miRNA (QIAGEN), synthetic miR-181b, or DCN siRNA, was transfected into SF using HiPerFect according to manufacturer protocols. After 48 hours cell culture supernatant was collected and DCN measured using ELISA. Total RNA was harvested and RT-qPCR for DCN mRNA performed. Untreated, or control miRNA, or antagomiR-181b was transfected into DF using HiPerFect. After 48 hours supernatant was collected and DCN measured using ELISA.

### TGF-β_1_ Stimulation of Dermal Fibroblasts and Measurement of Myofibroblast Differentiation by Flow Cytometry

Recombinant human TGF-β_1_ was used to stimulate site-matched NS and HSc fibroblasts in DMEM + 2% FBS at various concentrations for 48 hours. Control antagomiR or antagomiR-181b was transfected into HSc fibroblasts using HiPerFect. Cells were harvested using trypsin (Sigma-Aldrich) then permeabilized with saponin (Sigma-Aldrich) and stained using a phycoerythrin conjugated mouse monoclonal anti-human α-smooth muscle actin antibody (R&D Systems). Myofibroblasts were quantified using a FACSCanto II (BD Biosciences, San Jose, CA) flow cytometer and data analyzed using FACSDiva software (BD Biosciences).

### Statistical Analysis

All statistical analysis performed with Stata 10 (Stata Corportation, College Station, TX). Student’s t-test with Bonferroni correction, Wilcoxon signed-rank test, and Kruskal-Wallis rank test were used. P < 0.05 was considered significant.

## Results and Discussion

### DCN Expression is Lower in HSc as Compared to Site-matched NS, and Deep as Compared to Superficial Dermis

**Fig 1:**
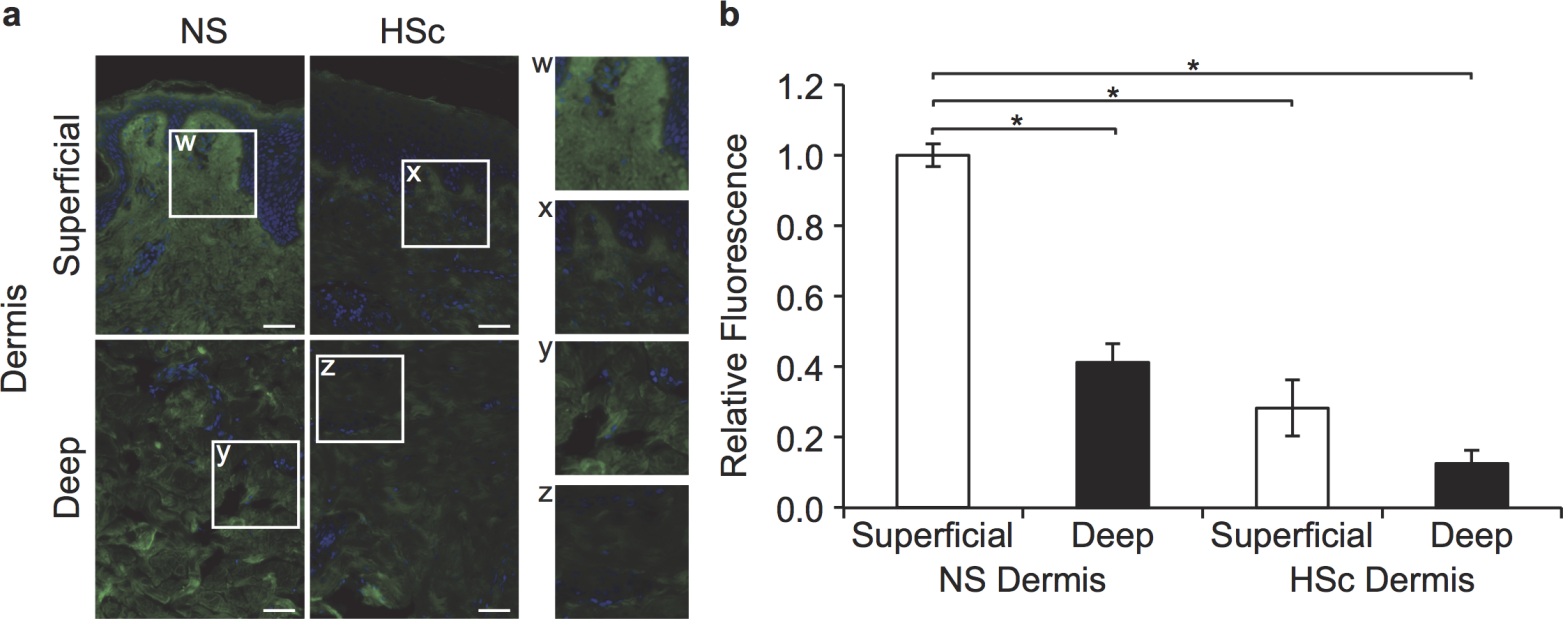
Immunohistochemical DCN expression in HSc and site-matched NS from burn patients. (a) Immunohistochemistry using a polyclonal goat anti-human DCN antibody and Alexa Fluor 488 secondary antibody (green fluorescence), and counterstained with DAPI (blue fluorescence) in representative site-matched sections of NS and HSc (scale bar = 50 μm). (b) Relative expression of DCN in matched superficial and deep NS and HSc sections was calculated from fluorescence using ImageJ (mean ± SEM, n = 4 patients, * P < 0.001).

To determine *in vivo* tissue DCN expression, immunohistochemistry was used to compare DCN in site-matched HSc and NS biopsies from burn patients, as shown in Fig 1A. DCN was significantly lower in HSc versus NS (P < 0.001), and deep versus superficial dermis in NS (P < 0.001), but not HSc (P = 0.055) (Fig 1B).

### DCN is Downregulated by miRNA in DF

HSc fibroblasts and DF produce less DCN than NS fibroblasts and SF *in vitro* [24,25]. One possible explanation for decreased DCN production in deep dermal and HSc fibroblasts is increased levels of miRNA targeting DCN. To test this hypothesis a DCN 3’UTR fluorescent reporter assay was created. Production of fluorescent protein DsRed2 as normalized to fluorescent protein EmGFP was significantly downregulated to 0.52 ± 0.06 versus a baseline of 1.0 ± 0.06 (P < 0.005) in DF but not in SF (P = 0.76), suggesting DCN regulation by increased miRNA in DF targeting the DCN 3’UTR in the DsRed2 construct.

### Several miRNA are Predicted to Regulate DCN in DF as Compared to SF

Potential miRNA regulating DCN were screened in silico and manually curated. Testing of miRNA predicted to interact with the DCN 3’UTR fragment and several others not predicted to interact was performed (S1A Fig). Because many miRNA were predicted, a PCR protocol (S1B Fig) used to screen a cDNA library for miRNA interactions [43] was modified for qPCR screening of mRNA 3’UTR-miRNA interactions. miR- 24, 181b, 421, 526b, and 543, had amplification efficiencies greater than 1.35 [41], as calculated using the formula in S1C Fig, and were further investigated.

**Fig 2:**
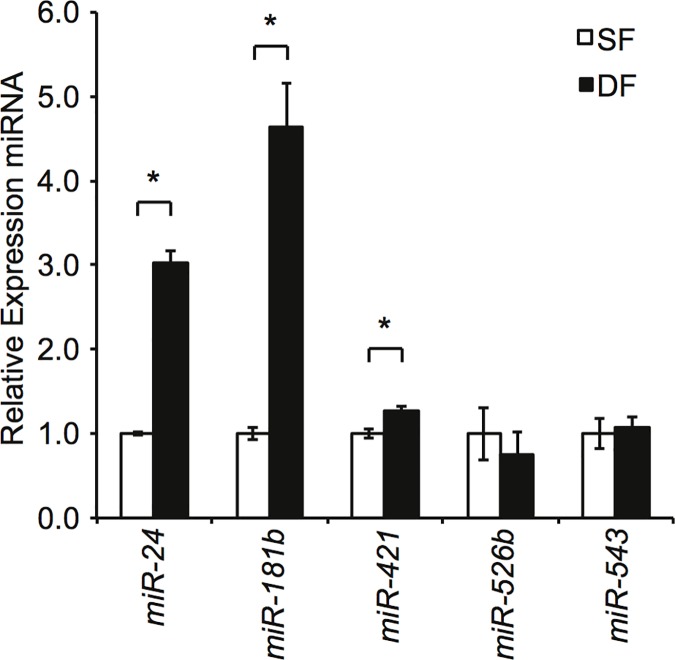
Evidence for the involvement of miRNA in *DCN* downregulation in DF. Total RNA was extracted from SF and DF cell culture after 48 hours and relative expression of selected miRNA quantitated using RT-qPCR (mean ± SEM, n = 3, * P < 0.05).

miR- 24, 181b, 421, 526b, and 543 in matched SF and DF were measured using RT-qPCR (Fig 2). Significantly higher levels of miR-24 (P < 0.05) and miR-181b (P < 0.05) were found in DF versus SF suggesting one might be responsible for decreasing DCN. In contrast, although miR-421 was expressed at statistically higher levels (P < 0.05) in DF, its magnitude was low so it was not investigated further.

### TGF-β_1_ Upregulates miR-181b Expression in Dermal Fibroblasts but CTGF does not

**Fig 3:**
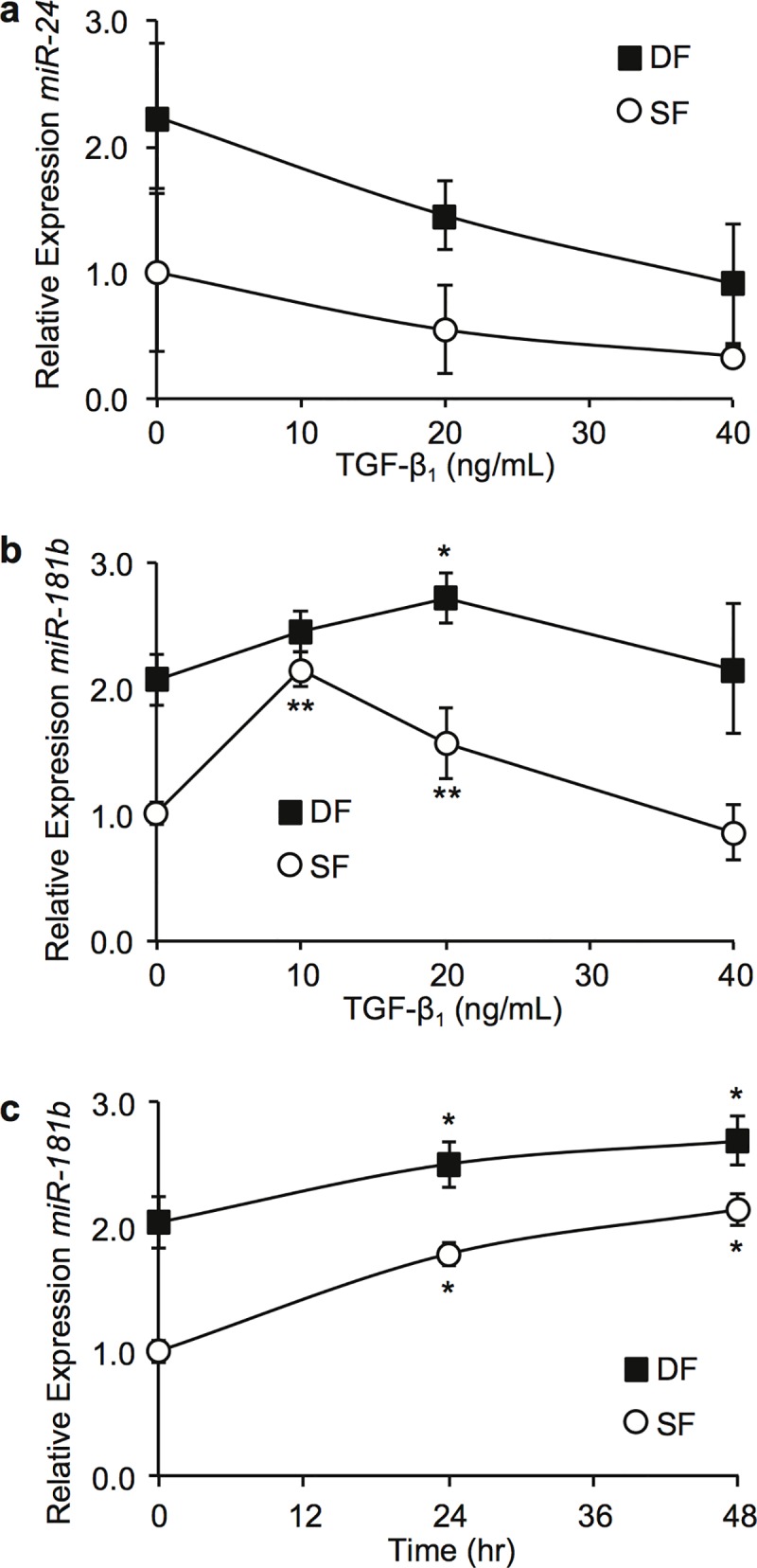
Regulation of miRNA expression by TGF-β_1_ in SF and DF. Cells were cultured in DMEM + 2% FBS with the indicated treatment protocols and total RNA extracted for RT-qPCR. (a) Dose-response curve showing relative expression of miR-24 for SF and DF cultured in increasing concentrations of TGF-β_1_ for 48 hours (mean ± SEM, n = 3). (b) Dose-response curve showing relative expression of miR-181b for SF and DF culutured in increasing concentrations of TGF-β_1_ for 48 hours (mean ± SEM, n = 3, * P < 0.05, ** P < 0.01). (c) Time-response curve showing relative expression of miR-181b for SF and DF at fixed concentrations of TGF-β_1_ (SF 10 ng/mL, DF 20 ng/mL) (mean ± SEM, n = 3, * P < 0.03).

Since TGF-β_1_ is a key profibrotic cytokine in HSc development [44], its effects on miR-24 and miR-181b in SF and DF were examined using RT-qPCR. miR-24 was downregulated by TGF-β_1_ in SF and DF in a dose-dependent manner (Fig 3A), in keeping with findings in myoblasts [45]. In contrast, miR-181b was significantly upregulated by a low concentration of TGF-β_1_ in SF (10 ng/mL) and DF (20 ng/mL) with a return to baseline by a high concentration of TGF-β_1_ (40 ng/mL) (Fig 3B), and the upregulation of miRNA-181b by TGF-β_1_ in SF (10 ng/mL) and DF (20 ng/mL) was a time-dependent manner (Fig 3C), similar to observations in hepatocytes [46]. A similar experiment using CTGF stimulation did not show changes in miR-181b expression. Based on these results miR-181b was selected for further investigation.

### miR-181b is Increased in HSc as Compared to Site-matched NS, and Deep as Compared to Matched Superficial Normal Dermis

**Fig 4:**
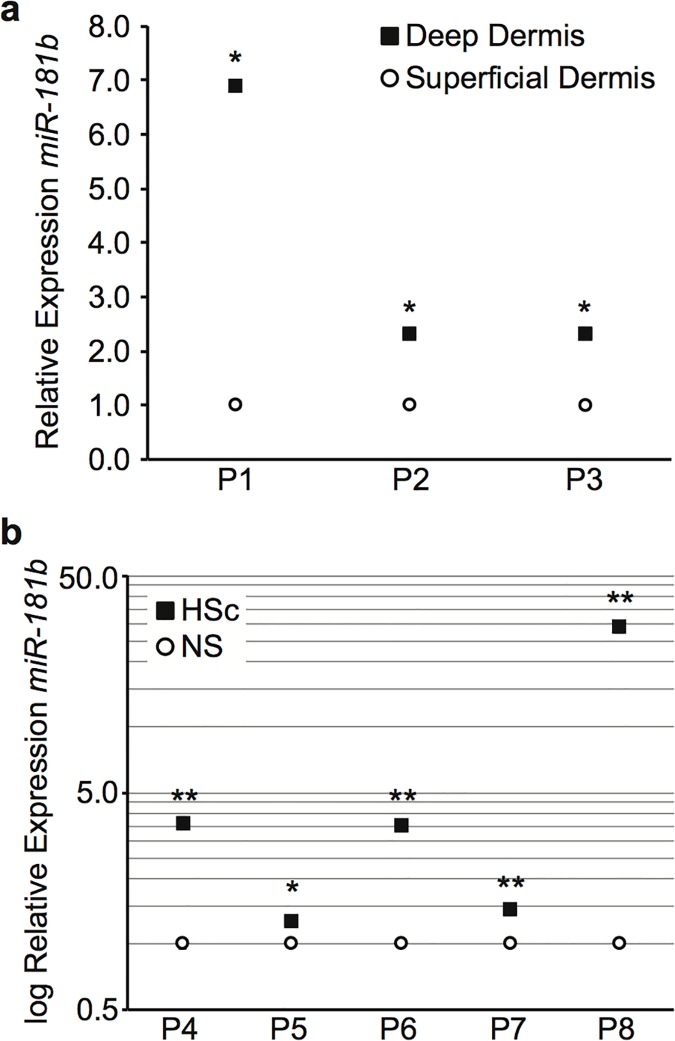
Relative expression of miR-181b in matched superficial and deep dermis and site-matched NS and HSc biopsies. Total RNA was extracted from tissue specimens using a chilled pestle and mortar and Trizol for relative quantitation using RT-qPCR. (a) Relative expression of miR-181b in matched superficial and deep dermis of NS (mean ± SEM, n = 3 samples per patient, * P < 0.001). (b) Relative expression of miR-181b in matched NS and HSc (mean ± SEM, n = 3 samples per patient, * P < 0.05, ** P < 0.01).

After identifying miR-181b as a potential downregulator of DCN *in vitro*, its expression *in vivo* in tissues known to express less DCN was examined using RT-qPCR of miRNA isolated from site-matched HSc and NS biopsies, and matched deep and superficial dermis. miR-181b was significantly increased in deep as compared to superficial dermis (Fig 4A), and HSc as compared to NS (Fig 4B).

### miR-181b Regulates DCN in Dermal Fibroblasts

**Fig 5:**
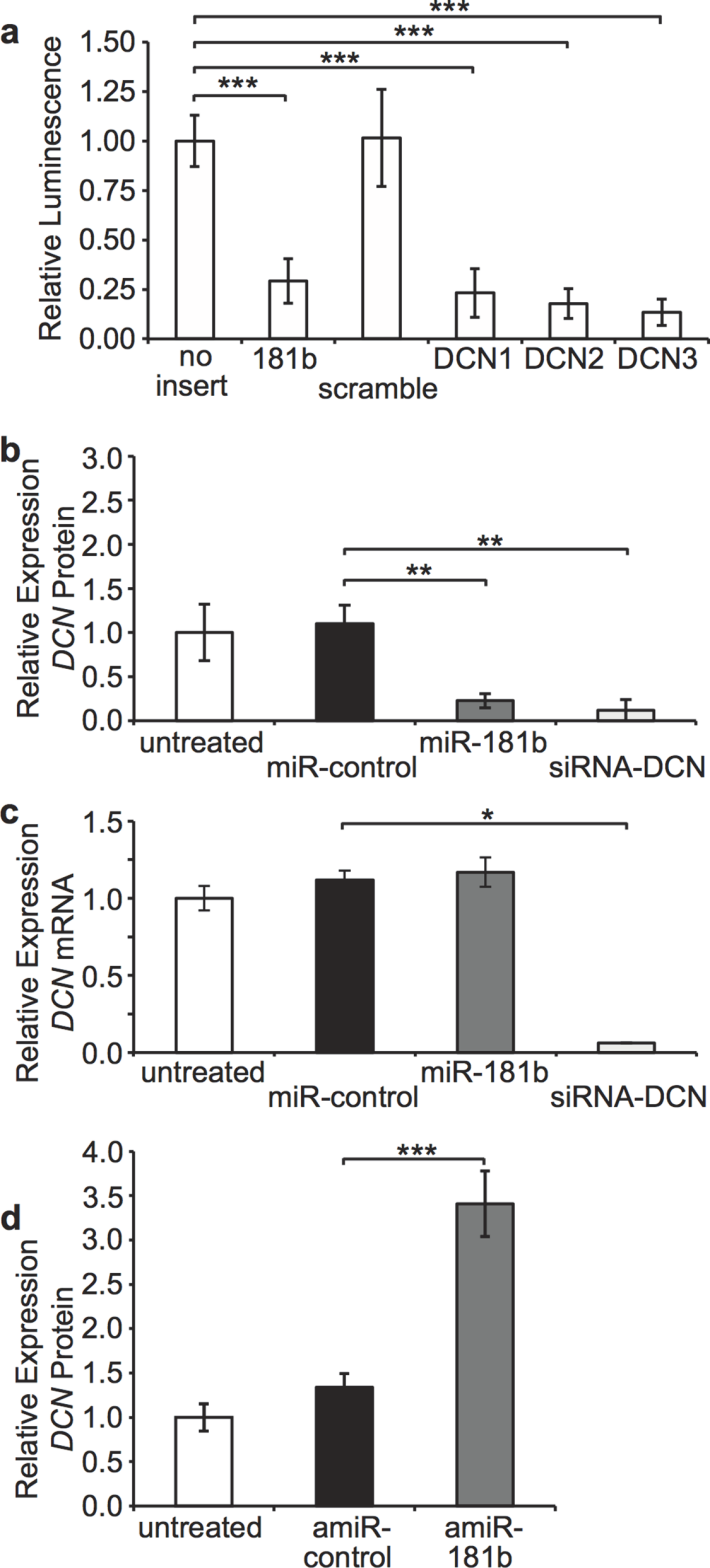
Regulation of DCN by miR-181b. HEK293A were cultured in DMEM + 2% FBS and transfected with pmirGLO constructs containing various miRNA binding sites and (a) relative fluorescence quantitated using a luminometer to determine relative knockdown by miR-181b (mean ± SEM, n = 4, *** P ≤ 0.01). SF were cultured in DMEM + 2% FBS and transfected with miR-control, synthetic miR-181b or siRNA-DCN and (b) DCN protein in supernatant was measured by ELISA (mean ± SEM, n = 3, ** P < 0.03), and (c) DCN mRNA was measured using RT-qPCR on total RNA (mean ± SEM, n = 3, * P < 0.05). (d) DF were cultured in DMEM + 2% FBS and transfected with antagomiR-control (amiR-control) or antagomiR-181b (amiR-181b) and DCN protein in supernatant was measured by ELISA (mean ± SEM, n = 3, *** P < 0.01).

To confirm predicted miR-181b binding sites from the DCN 3’UTR a series of dual luciferase reporter vectors based on pmirGLO were created and transfected into HEK293A cells (Fig 5A). There was no difference in regulation by miR-181b of reporters with no binding site or a scramble site (P = 0.96), however reporters with a perfect miR-181b site or one of three predicted miR-181b binding sites from the DCN 3’UTR (S2 Fig) were all significantly downregulated by miR-181b (P ≤ 0.01). One method to confirm miRNA regulation is to modulate miRNA levels and observe effects on its putative target [47]. Therefore, to confirm that miR-181b regulates DCN, synthetic miR-181b and antagomiR-181b were used to change miR-181b levels and changes in DCN measured in dermal fibroblasts. SF were transfected with a synthetic miR-181b mimic which significantly reduced DCN protein by ELISA (P < 0.03) (Fig 5B), similar to DCN siRNA (P < 0.02), but not DCN mRNA by RT-qPCR (Fig 5C). When DF were transfected with antagomiR-181b, DCN protein by ELISA was significantly increased (P < 0.01) (Fig 5D). DCN protein levels are expressed as fold changes to allow comparison despite variation between fibroblasts from different individuals.

### Blocking miR-181b Using antagomiR-181b Reverses TGF-β_1_ Induced Downregulation of DCN and Upregulation of Myofibroblast Differentiation in HSc Fibroblasts

**Fig 6:**
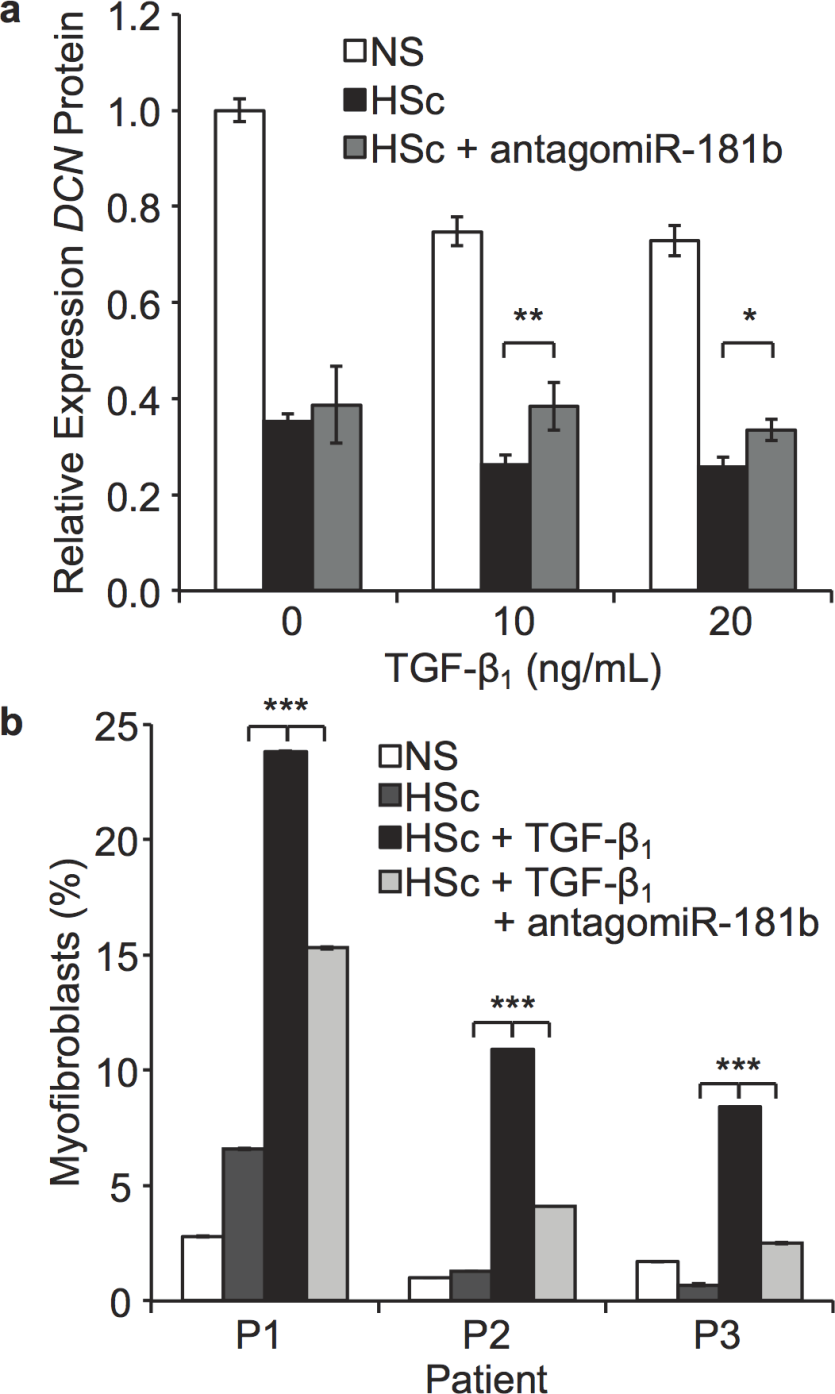
The effect of antagomiR-181b on TGF-β1 stimulated NS and HSc fibroblasts. (a) antagomiR-181b reversed DCN downregulation in HSc fibroblasts. Cells were stimulated by TGF-β1 at indicated concentrations and transfected with antagomiR-control or antagomiR-181b for 48 hours in DMEM + 2% FBS, and DCN protein was measured using ELISA on the supernatants (mean ± SEM, n = 3, * P < 0.02, ** P < 0.006). (b) antagomiR-181b reversed myofibroblast differentiation in HSc fibroblasts. Cells were stimulated by TGF-β1 10 ng/mL and transfected with antagomiR-control or antagomiR-181b for 48 hours in DMEM + 2% FBS then permeabilized and stained for α-smooth muscle actin and 10 000 cells per sample measured by flow cytometry (mean ± SEM, n = 3, *** P <0.03).

Based on prior results, blocking miR-181b might treat HSc, so this strategy was examined in matched NS and HSc fibroblasts treated with TGF-β_1_. As shown in Fig 6A, TGF-β_1_ stimulation significantly decreased DCN in both NS (P < 0.02) and HSc (P < 0.02) fibroblasts, and antagomiR-181b treatment reversed the decrease in DCN induced by TGF-β_1_ in HSc fibroblasts, returning DCN to baseline (P < 0.02). Again, DCN protein levels are expressed as fold changes to allow comparison despite inter-individual variation. As shown in Fig 6B, TGF-β_1_ stimulation significantly increased myofibroblast differentiation (mean 7.99 fold increase compared to baseline, P < 0.03), and antagomiR-181b treatment reversed this effect, significantly decreasing the number of myofibroblasts (mean 3.01 fold increase compared to baseline, P = 0.01).

Finding DCN was significantly downregulated in HSc as compared to NS, and deep as compared to superficial dermis, adds to previous observations that HSc fibroblasts produce significantly less DCN than NS fibroblasts [24], and confirms *in vivo* tissue patterns which match *in vitro* observations of decreased DCN production by DF [25]. Similarities between superficial and deep HSc, suggests it arises from DF. Observations that miR-181b was significantly upregulated in DF *in vitro* and deep dermis and HSc *in vivo*, suggests comparing SF and DF *in vitro* mimics their *in vivo* behavior. Furthermore, similarities in miR-181b expression between HSc and DF add to publications supporting the hypothesis that HSc fibroblasts arise from DF [25]. DCN expression was altered by miR-181b modulation, thus demonstrating miR-181b regulates DCN. Previous work on DCN regulation explored TGF-β_1_’s role in negatively regulating DCN in dermal fibroblasts via its promoter sequence [48,49], and our work demonstrating miR-181b, also induced by TGF-β_1_, downregulates DCN adds further insight into this complex regulatory relationship.

Members of the miR-181 family, originally described as specific to hematopoetic tissues [50], are found in many tissues including muscle [51] and endothelial cells [52], as well as cancers including multiple myeloma [53] and hepatocellular carcinoma [54]. Depending on context miR-181b either inhibits or promotes differentiation of cells by regulating various transcription factors. Ji et al. found miR-181b was upregulated in hepatic stem cells, embryonic livers, and hepatocellular carcinoma where it inhibited differentiation by targeting NLK, GATA6, and CDX2 transcription factors [54]. In contrast, in myoblasts miR-181b promotes differentiation by targeting the transcription factor Hox-A11 [55]. Arnold et al. found increased miR-181b was characteristic of the transition from stem to proliferating non self-renewing cells [56], and Shi et al. found miR-181b was a tumor repressor in gliomas [57]. As the only adult organ known to undergo regeneration, the liver is unique as compared to other human organs [58], and this may explain the alternate function of miR-181b. In any case, miR-181b does regulate key transcription factors determining cellular differentiation and function, and may also do so in dermal wound healing. Furthermore, if miR-181b activation seen in active muscle by Safdar et al. is due to repeated mechanical stress [51], one may speculate that this mechanism could be involved in the increased prevalence of HSc occurring in healing wounds over active joints [6]. Additionally, since miR-181b regulates a histone acetyl-transferase [53], it could also influence fibroblast epigenetics. It thus appears that many previously validated targets of miR-181b are transcription factors and signal transduction pathway components involved in regulating cellular behavior on a fundamental level, similar to the significant differences observed between NS and HSc fibroblasts, and suggesting that miR-181b regulation of DCN fits with the broad role DCN is already known to play in influencing cellular functions.

Knowing TGF-β levels are elevated in tissues following burn injury [8], suggests HSc fibroblasts exist in an environment of profibrotic TGF-β stimulation, and this promotes both DCN downregulation and myofibroblast differentiation. Based on these experiments it is possible to restore DCN production in TGF-β_1_ stimulated HSc fibroblasts to their basal level by blocking miR-181b and significantly reverse the differentiation of HSc fibroblasts into myofibroblasts. Although this does not revert HSc fibroblast DCN production to that of NS fibroblasts, this likely a result of epigenetic changes occurring during HSc formation. As scar matures, DCN expression returns to NS levels, suggesting events delaying normalization contribute to the prolonged abnormalities seen in HSc. Blocking miR-181b removed this pathway of DCN downregulation and normalized DCN production. Perhaps this could restore balance between profibrotic (e.g. TGF-β_1_) and antifibrotic (e.g. DCN) factors, thus encouraging scar maturation rather than fibroproliferation, and serving as a potential therapy for HSc.

The observations of increases in basal miR-181b expression in HSc and DF suggest miR-181b may serve an epigenetic role [59] in altered DCN expression levels in these cells and tissues. Given miR-181b’s previously described roles in a wide variety of tissues and its regulation of several transcription factors and a histone acetyl-transferase, one may speculate that it could serve a broad regulatory role. Experiments to determine if miR-181b downregulates other antifibrotic signaling molecules or is upregulated by additional profibrotic cytokines would help further delineate its role in wound healing and fibrosis. And in fact, in silico prediction algorithms suggest that miR-181b targets several other key factors involved in wound healing (S4 Table).

## Conclusion

Given these findings, antagomiR-181b is a potential therapy for HSc and may either prevent its occurrence or accelerate its resolution through restoring targets of miR-181b, including DCN.

## Supporting Information

S1 Fig miRNA qPCR screening. To compare the binding of various miRNA to the 3’ UTR of interest a miRNA qPCR screening protocol was created. A forward primer homologous to the plasmid pDNR-LIB-DCN just 5’ of the DCN 3’ UTR was created. The various miRNA of interest were created as reverse primers, and a scramble sequence and sequence homologus to pDNR-LIB-DCN sequence just 3’ of the DCN 3’ UTR were created as negative and positive control primers. To determine the relative efficiency (E) of the miRNA as a reverse primer the equation in S1c was used. The efficiency (E) of the qPCR reaction was set as 2 for the positive control, and the same quantity (Q) of pDNR-LIB-DCN was used as template for all reactions. This allowed the relative efficiency of the miRNA as a primer to be calculated. Those miRNA with relative efficiencies greater than 1.35 [41] were considered as interacting with the 3’ UTR and the otherwise were not. The efficiency of the negative control primer was verified to be less than 1.35 as expected. (a) Results of miRNA qPCR screening suggest that miR- 24, 181b, 421, 526b, and 543 potentially target the DCN 3’UTR (* P < 0.005, ** P < 0.0005). (b) PCR cycle parameters allowing nonspecific binding of mature miRNA at physiologic temperatures. (c) Derivation of the efficiency equation to determine interacting and non-interacting miRNA.
(EPS)

S2 Fig DCN 3’UTR (NCBI accession NM_001920.3) showing predicted miR-181b binding sites in bold with potential base pair alignment. (EPS)

S1 Table Patient information. (DOC)

S2 Table Primer sequences used for (a) miRNA qPCR screening, (b) Sticky-end PCR, and (c) RT-qPCR of mRNA. (DOC)

S3 Table Sequences used for miRNA binding sites in pmirGLO dual luciferase reporter plasmid. (DOC)

S4 Table Select in silico predicted miR-181b targets involved in fibrosis and wound healing using TargetScan [38]. (DOC)
