# Supplementary material for: MicroRNA 181b Regulates Decorin Production by Dermal Fibroblasts and May Be a Potential Therapy for Hypertrophic Scar
Source: PLoS One. 2015 Apr 2;10(4):e0123054. doi: 10.1371/journal.pone.0123054 (PMC4383602; doi:10.1371/journal.pone.0123054)
Supplement: S1 Table — (DOC) [file pone.0123054.s003.doc]

**Table S1. Patient information.**

| Patient | Sex | Age | Total Body Surface Area Burn (%) | Injury |
| --- | --- | --- | --- | --- |
| P1 | Male | 23 | 0 | None |
| P2 | Male | 23 | 0 | None |
| P3 | Female | 37 | 0 | None |
| P4 | Male | 23 | 18 | Burn |
| P5 | Male | 37 | 35 | Burn |
| P6 | Male | 46 | 20 | Burn |
| P7 | Male | 42 | 50 | Burn |
| P8 | Male | 27 | 40 | Burn |
