# Supplementary material for: MicroRNA 181b Regulates Decorin Production by Dermal Fibroblasts and May Be a Potential Therapy for Hypertrophic Scar
Source: PLoS One. 2015 Apr 2;10(4):e0123054. doi: 10.1371/journal.pone.0123054 (PMC4383602; doi:10.1371/journal.pone.0123054)
Supplement: S2 Table — (DOC) [file pone.0123054.s004.doc]

**Table S2. Primer sequences used for (a) miRNA qPCR screening, (b) Sticky-end PCR, and (c) RT-qPCR of mRNA.**

| **Primer** | **Sequence 5’-3’** |
| --- | --- |
| **a** | |
| anchor | ATGTGTCTACGTGCGCTCTG |
| +ve Control | CCATCTGGATTTGTTCAGAACGCTCGGTTGCC |
| -ve Control | TAGCACCATTTGAAATCAGTGTT |
| miR-24 | TGGCTCAGTTCAGCAGGAACAG |
| miR-181b | AACATTCATTGCTGTCGGTGGGT |
| miR-191 | CAACGGAATCCCAAAAGCAGCTG |
| miR-218 | TTGTGCTTGATCTAACCATGT |
| miR-299-3p | TATGTGGGATGGTAAACCGCTT |
| miR-421 | ATCAACAGACATTAATTGGGCGC |
| miR-491-3p | CTTATGCAAGATTCCCTTCTAC |
| miR-526b | CTCTTGAGGGAAGCACTTTCTGT |
| miR-543 | AAACATTCGCGGTGCACTTCTT |
| miR-590-3p | TAATTTTATGTATAAGCTAGT |
| miR-875-3p | CCTGGAAACACTGAGGTTGTG |
| **b** | |
| DCN 3’UTR A | GGCCGCTTCTCAAGAAAGCCCTCATT |
| DCN 3’UTR B | CGTTCTCAAGAAAGCCCTCATT |
| DCN 3’UTR C | GGCCGCAGCTTTACTAAATATTGACATATATATTTACT |
| DCN 3’UTR D | CGAGCTTTACTAAATATTGACATATATATTTACT |
| EmGFP A | AATTCGCCACCATGGTGAGCAAG |
| EmGFP B | CGCCACCATGGTGAGCAAG |
| EmGFP C | GGCCGCATCAAGCTTCTCGAG |
| EmGFP D | GCATCAAGCTTCTCGAGTTACTTGTACAG |
| **c** | |
| DCN F | GGCTTCTTATTCGGGTGTGA |
| DCN R | CAGAGCGCACGTAGACACAT |
| HPRT1 F | CTCCGTTATGGCGACCC |
| HPRT1 R | CACCCTTTCCAAATCCTCAG |
