# Supplementary material for: MicroRNA 181b Regulates Decorin Production by Dermal Fibroblasts and May Be a Potential Therapy for Hypertrophic Scar
Source: PLoS One. 2015 Apr 2;10(4):e0123054. doi: 10.1371/journal.pone.0123054 (PMC4383602; doi:10.1371/journal.pone.0123054)
Supplement: S3 Table — (DOC) [file pone.0123054.s005.doc]

**Table S3. Sequences used for miRNA binding sites in pmirGLO dual luciferase reporter plasmid.**

| **Plasmid** | **Sequence 5’-3’** |
| --- | --- |
| pmirGLO | No insert |
| pmirGLO-181b | ACCCACCGACAGCAATGAATGTT |
| pmirGLO-scramble | TGGGCGTATAGACGTGTTACAC |
| pmirGLO-DCN1 | AACCTAACTGCAATGTGGATGTT |
| pmirGLO-DCN2 | CATTACTGGTAAAGCCTCATTTGAATGTG |
| pmirGLO-DCN3 | TTATGTCATCTATGTTGAATGTA |
