# Supplementary material for: MicroRNA 181b Regulates Decorin Production by Dermal Fibroblasts and May Be a Potential Therapy for Hypertrophic Scar
Source: PLoS One. 2015 Apr 2;10(4):e0123054. doi: 10.1371/journal.pone.0123054 (PMC4383602; doi:10.1371/journal.pone.0123054)
Supplement: S4 Table — (DOC) [file pone.0123054.s006.doc]

**Table S4. Select *in silico* predicted miR-181b targets involved in fibrosis and wound healing using TargetScan [38].**

| **Target Gene Symbol** | **Target Gene Name** |
| --- | --- |
| IL2 | Interleukin 2 |
| TGFBRAP1 | Transforming growth factor beta receptor associated protein 1 |
| TIMP3 | Tissue inhibitor of metalloproteinase 3 |
| HSP90B1 | Heat shock protein 90kDa beta 1 |
| IL1A | Interleukin 1 alpha |
| BMP3 | Bone morphogenetic protein 3 |
| TGFBR1 | Transforming growth factor beta receptor 1 |
| SIRT1 | Sirtuin 1 |
| PLAU | Plasminogen activator urokinase |
| SMAD7 | SMAD family member 7 |
| TGFBR2 | Transforming growth factor beta receptor 2 |
| VCAN | Versican |
| SMAD2 | SMAD family member 2 |
| FGFR3 | Fibroblast growth factor receptor 3 |
